# Preparation of wood-like structured copper with superhydrophobic properties

**DOI:** 10.1038/srep18328

**Published:** 2015-12-11

**Authors:** Tianchi Wang, Guiju Liu, Jian Kong

**Affiliations:** 1 Nanjing University of Science and Technology, School of Materials Science and Engineering, Nanjing 210094, China

## Abstract

Here, we report a method to use natural wood lauan as a template to fabricate superhydrophobic biomorphic copper on a carbon substrate (Cu/C). First, a carbon substrate with the microstructure of lauan was obtained by sintering lauan in an oxygen-free environment. A biomorphic Cu/C material was then obtained by immersing this carbon substrate into a Cu(NO_3_)_2_ solution and sintering. Finally, the hydrophobicity of the products obtained was investigated. The Cu/C retained the microstructure of the wood well. It exhibited excellent superhydrophobicity after it was modified with fluorine silane. The water contact angle of this modified Cu/C reached 160°.

Wettability is an important feature of solid surfaces, and it is determined by the chemical composition and microstructure of the surfaces^1^. Over the past decade, researchers have paid more and more attention to superhydrophobic surfaces with water contact angles (CAs) higher than 150° and sliding angles (SAs) lower than 10°. Superhydrophobic surfaces have many potential applications in daily life and industrial production^2^,^3^,^4^. Examples include self-cleaning windows, windshields, exterior paints for buildings and ships, utensils, roof tiles, textiles, solar panels, and micro- and nanofluidic applications requiring reduced in fluid flow drag. In general, two methods can be used to prepare a superhydrophobic surface. The first method is to create roughness on a hydrophobic material surface. The second method is to modify a low-surface-energy material on a rough surface.

As an important engineering material, copper is widely used in many industrial applications. The development of Cu surfaces with superhydrophobic properties can increase the corrosion resistance and extend the service life of devices fabricated using this material. It can also expand the range of traditional applications of these devices. Superhydrophobic Cu surfaces are usually prepared by constructing rough surface structures and coating them with low-surface-energy materials that often contain fluorine^5^,^6^. Many methods have been reported for constructing rough Cu surfaces, such as electroless deposition^7^,^8^, chemical etching^9^,^10^, electroplating^11^, and the solution-immersion process^12^, etc. In addition, some researchers prepared superhydrophobic materials with special methods^13^,^14^,^15^,^16^,^17^. Deng *et al.* chose candle soot as a template for a transparent robust coating; after sintering and silanization, the coating was superamphiphobic. Bayer *et al.* also prepared robust superhydrophobic surfaces from small diffusion flame treatment of hydrophobic polymers.

Some plant leaves have superhydrophobic capabilities, such as the lotus leaf, rice leaf, rose petals, *Nasturtium* leaf, and peanut leaf, because of the combination of a hierarchical structures with a low-surface-energy wax coating^18^. The main purpose of constructing a rough Cu surface is to imitate the microstructure found on such plant leaves. However, we cannot precisely mimic the fantastic surface structures of hydrophobic leaves, even by using the highest levels of technology. This limits further improvement of the superhydrophobic properties of materials.

Under normal circumstances, natural materials such as wood, cotton, and jute, which are superior to synthetic materials, possess highly evolved structures with amazing combinations of functions and capabilities. Inspired by nature, researchers have selected certain biostructures from different plant and animal species to use as templates for synthesizing various desired ceramics such as SiC, TiC, Al_2_O_3_, and SnO_2_. These products retain the microstructures of the original, natural materials, which are the key factors that provide the materials with excellent new properties^19^,^20^.

Recently, we used lotus leaf, rice leaf, and indicalamus leaf as templates for sintering and employed the modified fluorine silane (FAS) method to fabricate biomorphic superhydrophobic Cu and ZnO surfaces with contact angles above 160° 21,22. This high value was achieved because of the rough microstructure of the leaf surface. According to the Cassie model theory^23^, an air film is formed between water droplets and the substrate surface. Thus, the water droplets have few points of contact with the sample, thereby reducing the contact area.

Wood has a rich, rough, and porous surface structure, with pore sizes ranging from several microns to tens of microns. After sintering in an oxygen-free environment, the mixed biopolymers in wood will decompose into carbon and gases. This gives rise to porous carbon with the morphology derived from the wood template^19^,^24^. Porous carbon has many favorable characteristics such as good electromagnetic shielding properties, stable coefficient of friction, excellent far-infrared properties and high damping capacity. These outstanding properties can be attributed to the rational structures of wood. A porous surface structure may also exhibit superhydrophobicity because water droplets on the surface will have a small contact area. The porous surface can trap the air, and thus the water droplets on it cannot come into contact with the bottom. The porosity of wood makes it possible to prepare superhydrophobic materials. Therefore, in this study, we used natural wood lauan as a template to fabricate superhydrophobic biomorphic Cu on a carbon substrate (Cu/C) mainly through sintering the wood template and chemical modification. Then, the performance of the obtained surface was investigated. If this product can inherit a porous structure similar to that of original wood, it may also have superhydrophobic properties. The main purpose of constructing the rough Cu surface is to imitate the microstructure found on the lauan wood. Traditional preparation methods cannot precisely mimic the fantastic surface structures of the woods, even by using the most advanced technology and equipment. Our preparation method is simple and easy to control, providing a new idea for the preparation of superhydrophobic materials.

## Results

### Phase analysis

**Figure 1:**
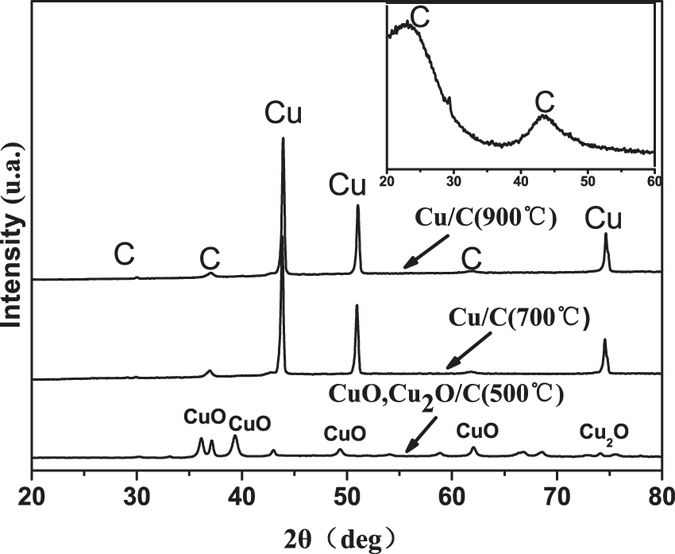
XRD patterns of biomorphic carbon, CuO, Cu_2_O/C, and Cu/C derived from the lauan.

X-ray diffraction (XRD) patterns of the materials derived from the lauan wood are presented in Fig. 1. The XRD pattern of the sample prepared by sintering the lauan to 600 °C is shown in the inset of Fig. 1; two broad peaks appeared at 23° and 43°, which are the salient features of non-graphite. These features are present because the organics were decomposed into carbon and gas during sintering, whereas it can only be converted into an amorphous structure at a low temperature such as 600 °C. Then, the biomorphic carbon was immersed into a Cu(NO_3_)_2_ solution and sintered at several different temperatures. When the sintering temperature reached 500 °C, crystalline CuO and Cu_2_O peaks appeared. The formation of CuO and Cu_2_O depends mainly on the decomposition of Cu(NO_3_)_2_ and the reduction reaction between C and CuO; the following two chemical reactions provide an explanation: 2Cu(NO_3_)_2_ = 2CuO + 4NO_2_↑ + O_2_↑ and C + 4CuO = 4Cu_2_O + C↑. The reactions only occurred for copper oxide overdose and at low temperatures. Upon increasing the sintering temperature, copper peaks began to emerge, and the degree of crystallization for Cu increased. In addition, carbon appeared in a crystalline state with the appearance of weak sharp peaks.

### Surface morphology and composition analysis

**Figure 2:**
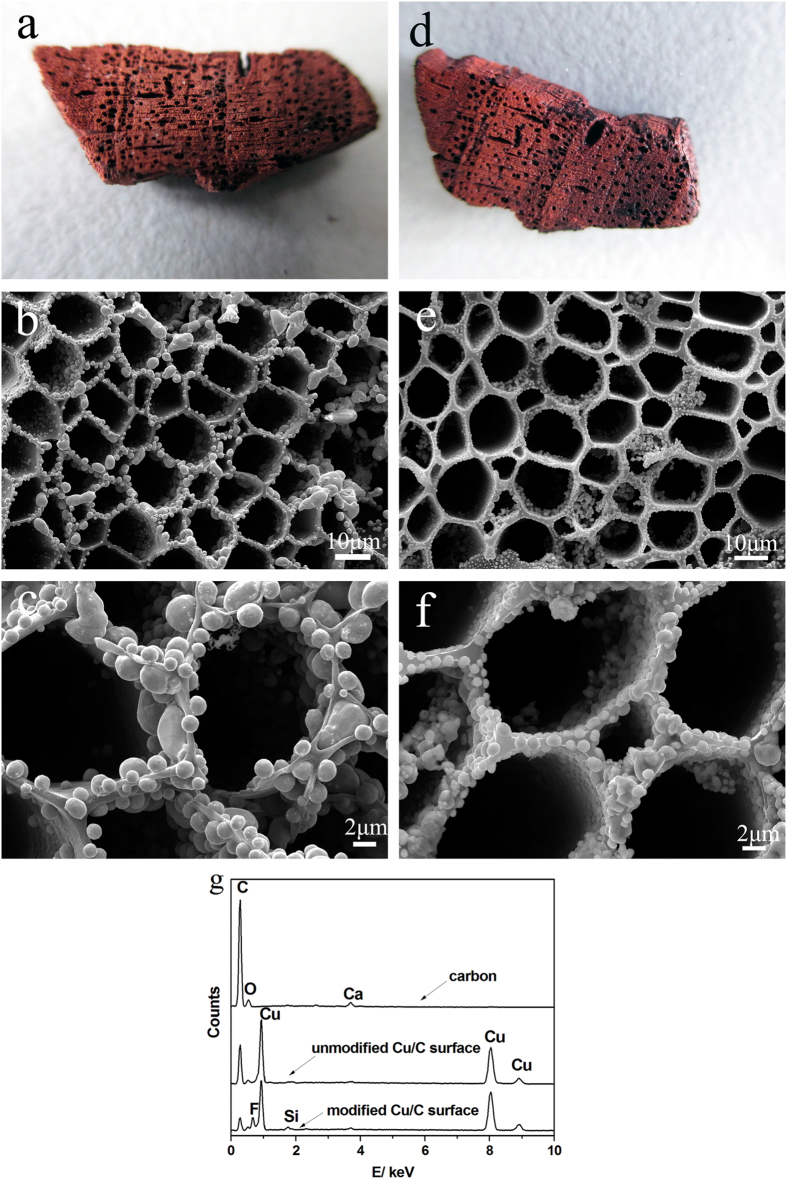
Full-scale images of (**a**) unmodified Cu/C and (**d**) modified Cu/C; SEM images of the surfaces of (**b**) unmodified biomorphic Cu/C and (**e**) modified biomorphic Cu/C; and (**c**) and (**f**) enlarged images of (**b**) and (**e**), respectively; (**g**) EDS spectra of the surfaces of biomorphic carbon, unmodified Cu/C, and modified Cu/C.

Figure 2 shows the surface morphology of the Cu/C obtained by immersing the carbon into a Cu(NO_3_)_2_ solution and sintering it at 900 °C before and after surface modification with FAS. The surface of the original Cu/C sample was very coarse, with a rich pore structure, as observed in Fig. 2a,d. The Cu/C surfaces maintained the macroscale structure of the lauan. In addition, the color of the carbon changed from black to red, which indicated that Cu covered the surface of the biomorphic carbon.

Figure 2b,c,e,f show the scanning electron microscope (SEM) images and magnified images of the original Cu/C and modified Cu/C, respectively. As observed in Fig. 2b,c, the unmodified Cu/C surface consisted of a honeycomb-like microstructure, replicating the morphology of the lauan. This result occurred because the organics in the lauan decomposed into carbon and gases during sintering in the oxygen-free environment, yielding biomorphic carbon with a structure derived from the lauan template, and the microstructure of lauan was not damaged during sintering. Furthermore, we can see that Cu was deposited onto the pore surface and interior, with spherical shapes of various sizes. The dimensions of the spheres were several microns to several hundred nanometers. The ball-like microscopic structures over the entire substrate can enhance the surface roughness, which would be helpful for preparing a superhydrophobic surface. As observed in Fig. 2e,f, we can also see that the surfaces of the modified Cu/C closely conform to the lauan morphology and the shape of the Cu was not damaged. The SEM results indicate that the surfaces of the samples had hierarchical micro- and nano-surface structures and that the pores were filled with air, which undoubtedly reduced the contact area between the water drops and the substrates. Furthermore, we can make the following speculation: Cu covered the surface of biomorphic carbon. First, a Cu(NO_3_)_2_ coating formed on the surface of the biomorphic carbon after immersing the carbon into the Cu(NO_3_)_2_ solution and drying. Then, during sintering with increasing temperature, the Cu(NO_3_)_2_ coating decomposed into CuO, which was followed by a reaction between CuO and the carbon substrate to form the Cu coating *in situ*.

Figure 2g shows the energy dispersive spectrometer (EDS) spectra of the biomorphic carbon, unmodified Cu/C, and modified Cu/C. The results indicate that C, O, and other trace elements were present in the biomorphic carbon, whereas clear Cu peaks appeared for the unmodified Cu/C surface. This result was observed because with the increase of temperature during sintering, the Cu(NO_3_)_2_ solution was completely decomposed, and then, a reduction reaction occurred with the carbon. Then, after modification by FAS, the surface of the sample exhibited an F peak, which indicates that FAS was successfully grafted onto the substrate.

**Figure 3:**
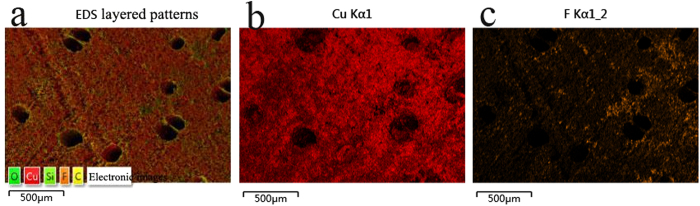
Elemental distribution of Cu and F on the modified Cu/C surface: (**a**) SEM image of the scanning area, (**b**) distribution of Cu (red areas), and (**c**) distribution of F (yellow areas).

Figure 3 shows the distribution states of Cu (Fig. 3b) and F (Fig. 3c) on the modified Cu/C surface (Fig. 3a). The red and yellow regions represent Cu and F, respectively. The images reveal that after the two-step treatment with Cu(NO_3_)_2_ solution and FAS, Cu and F were almost evenly distributed on the surface of the biomorphic carbon. It is supposed that Cu and FAS covered the biomorphic carbon similar to a coating.

### Fourier transform infrared (FT-IR) analysis

**Figure 4:**
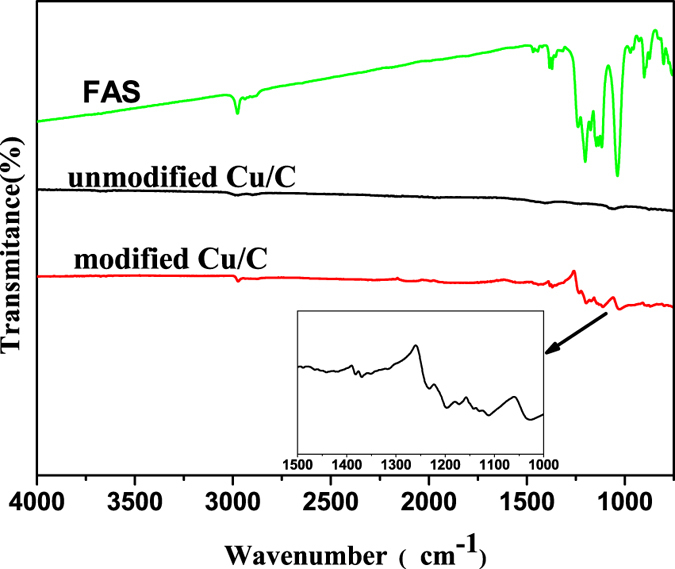
FT-IR spectra of FAS, unmodified Cu/C, and modified Cu/C; the inset shows the enlarged spectrum between 1000 and 1500 cm^−1^.

The chemical bonding in the modified Cu/C sample was investigated using infrared spectroscopy over the wavenumber range of 750 to 4000 cm^−1^. The results are presented in Fig. 4, and the insets show enlarged graphs of the corresponding curves in the range of 1000 to 1500 cm^−1^. The IR spectra of the modified Cu/C sample reveals that the C–H stretching peak at 2976 cm^−1^, corresponding to methyl or methylene groups, and the peak at 1467 cm^−1^, corresponding to CH_3_ or CH_2_ deformations disappeared, which demonstrates that the FAS was fully hydrolyzed. As compared to unmodified Cu/C, the surface after modification with FAS is characterized by three new peaks at 1369, 1197, and 1108 cm^−1^. These peaks correspond to stretching vibrations in CF_3_, CF_2_, and Si–O–Si, respectively. This finding indicates that after chemical modification, FAS formed a self-assembled monolayer on the Cu/C surface through hydrolysis and polycondensation. The hydrophobic groups (−CF_3_, with a surface energy of 6.7 mJ/m^2^; −CF_2_, with a surface energy of 18 mJ/m^2^) of FAS are critical to lowering the surface free energy of the carbon ceramic.

### Superhydrophobic properties

**Figure 5:**
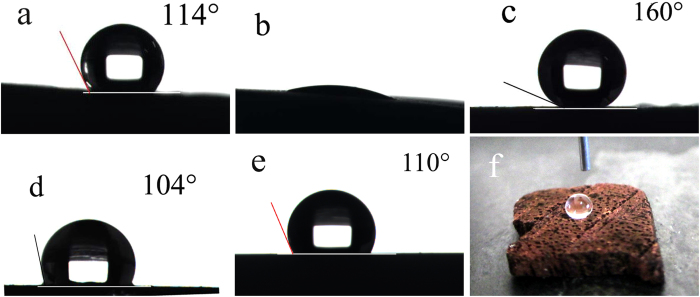
Images of the water CA of the (a) biomorphic carbon derived from lauan (114°), (b) unmodified Cu/C (~0°), (c) modified Cu/C (160°), (d) modified smooth Cu (104°), and (e) modified smooth carbon (110°). (**f**) Macro-photograph of droplet on modified Cu/C.

Figure 5 shows the water contact angles on the different surfaces. On the unmodified biomorphic carbon surface derived from lauan wood, the water CA is 114° (Fig. 5a); this range of contact angle can be described as representing a hydrophobic property, which thanks to the porous structure of the carbon, reduces the contact area between the droplets and substrate. However, on the unmodified Cu/C surfaces, the water cannot form a droplet; instead, the water spread, as observed in Fig. 5b. The water CA of the unmodified Cu/C surfaces is almost 0° because Cu is hydrophilic, indicating that the chemical composition of the surface can greatly affect the hydrophobicity. Figure 5c shows a superhydrophobic surface with a contact angle of 160° after the Cu/C surface was modified by FAS. However, it should be noted that the water CA is only 104° and 110° on a modified smooth Cu surface and smooth carbon surface, respectively (with no rough structures), as shown in Fig. 5d,e. This finding indicates that the surface roughness plays an important role in the fabrication of superhydrophobic surfaces. In Fig. 5f, we can also see that the water droplet is almost spherical on the modified Cu/C surface.

The Cassie-Baxter model was applied to describe the superhydrophobic characteristics of the sample surface^23^ using the equation cosθ^*^ = (1 − *f*) (*cosθ* + 1) − 1, where θ* and *θ* are the CAs on rough and smooth carbon surfaces, respectively, and f corresponds to the area in contact with the trapped air. According to the above results, the water CA on the modified Cu/C surfaces was 160°, whereas on the modified smooth carbon, it was only 110°. Inserting these CAs into the formula, the value of f was calculated to be 0.91, which indicates that only 9% of the modified Cu/C surface was in direct contact with the water; more than 90% was covered by air. Water on such a surface is similar to that on an air cushion; only a few points of contact are made with the convex top, making it appear suspended on the surface, resulting in a high surface contact angle.

## Methods

### Sample Preparation

Dried lauan was used as the biological plant template. The samples were first sintered in an oxygen-free environment at a heating rate of 2 °C/min to 600 °C, after which they were kept warm for 1 h to obtain pyrolytic carbon^19^. Then, these biomorphic carbon samples were immersed in a Cu(NO_3_)_2_ solution (30 wt% aqueous solution) for 60 min under a vacuum environment. Next, the samples were heat treated in an oven at 80 °C for 2 h. After drying, the samples were sintered in Ar at 500 °C, 700 °C, and 900 °C in a tube furnace (heating at 2 °C/min to 200 °C) and then to 500 °C, 700 °C, and 900 °C (at a rate of 3 °C/min). During the high-temperature sintering, copper nitrate was decomposed and reduced to Cu. Subsequently, the Cu/C samples were put into small test tubes; fluorine silane and isopropanol were added dropwise (at a volume ratio of 1:5) using disposable plastic head droppers. The Cu/C samples were immersed in this fluorine silane solution for 3 to 5 days at room temperature. After dipcoating of FAS coating, the samples were placed in air to dry naturally.

### Characterization

The crystalline structure of the samples was investigated using XRD (D8 Advance, Germany). The surface morphology was examined using SEM (Quant 250FEG), and the corresponding elemental distributions on the surface were determined using EDS. The chemical bond structure was analyzed using a FT-IR spectrometer (Nicolet IS-10, America). The water CAs were measured with a contact angle measurement instrument (JC2000D2, Shanghai Zhongchen Digital Technology Apparatus Co., Ltd) with distilled water droplet volumes of 4 *μ*L. The average CA values were determined based on measurements at more than five different positions for each sample.

## Additional Information

**How to cite this article**: Wang, T. *et al.* Preparation of wood-like structured copper with superhydrophobic properties. *Sci. Rep.* **5**, 18328; doi: 10.1038/srep18328 (2015).
